# Polyalthia Clerodane Diterpene Potentiates Hypoglycemia via Inhibition of Dipeptidyl Peptidase 4

**DOI:** 10.3390/ijms20030530

**Published:** 2019-01-27

**Authors:** Po-Kai Huang, Shian-Ren Lin, Jirawat Riyaphan, Yaw-Syan Fu, Ching-Feng Weng

**Affiliations:** 1 Department of Life Science and Institute of Biotechnology, National Dong Hwa University, Hualien 97401, Taiwan; kevin7699402@hotmail.com (P.-K.H.); d9813003@gms.ndhu.edu.tw (S.-R.L.); 810254005@gms.ndhu.edu.tw (J.R.); 2 Departmental of Biomedical Science and Environmental Biology, Kaoshiung Medical University, Kaoshiung 80708, Taiwan; m805004@kmu.edu.tw

**Keywords:** dipeptidyl peptidase 4, hyperglycemia, natural compound inhibitors, molecular docking, clerodane diterpene 16-hydroxycleroda-3,13-dien-15,16-olide

## Abstract

Serine protease dipeptidyl peptidase 4 (DPP-4) is involved in self/non-self-recognition and insulin sensitivity. DPP-4 inhibitors are conventional choices for diabetic treatment; however, side effects such as headache, bronchus infection, and nasopharyngitis might affect the daily lives of diabetic patients. Notably, natural compounds are believed to have a similar efficacy with lower adverse effects. This study aimed to validate the DPP-4 inhibitory activity of clerodane diterpene 16-hydroxycleroda-3,13-dien-15,16-olide (HCD) from *Polyalthia longifolia*, rutin, quercetin, and berberine, previously selected through molecular docking. The inhibitory potency of natural DPP-4 candidates was further determined by enzymatic, in vitro Caco-2, and ERK/PKA activation in myocyte and pancreatic cells. The hypoglycemic efficacy of the natural compounds was consecutively analyzed by single-dose and multiple-dose administration in diet-induced obese diabetic mice. All the natural-compounds could directly inhibit DPP-4 activity in enzymatic assay and Caco-2 inhibition assay, and HCD showed the highest inhibition of the compounds. HCD down-regulated LPS-induced ERK phosphorylation in myocyte but blocked GLP-1 induced PKA expression. For in vivo tests, HCD showed hypoglycemic efficacy only in single-dose administration. After 28-days administration, HCD exhibited hypolipidemic and hepatoprotective efficacy. These results revealed that HCD performed potential antidiabetic activity via inhibition of single-dose and long-term administrations, and could be a new prospective anti-diabetic drug candidate.

## 1. Introduction

Type 2 diabetes mellitus (TII DM) is a chronic disease that occurs in 366 million people worldwide and the effects of TII DM result in cardiovascular disease, obesity, eye problems, kidney problems, problems with the feet, nerves or microvascular complications, damage to large blood vessels, as well as brain, legs and macrovascular complications [1]. According to statistics in 2016, the disability-adjusted-life-years (DALYs) rate of diabetes is 1887.6/100,000, which has become a burden for all of society [2]. Medication is the main regimen for treating diabetes and there are numerous varieties of hypoglycemic agents available, such as sulfonylurea, α-glucosidase inhibitors, Na^+^/glucose co-transporter inhibitors, and biguanides [3]. Noticeably, inhibitors of dipeptidyl peptidase 4 (DPP-4) have an alternative impact on diabetic strategy.

DPP-4 (also named as adenosine deaminase complexing protein 2, ADCP 2) is a serine exopeptidase that is a cleaved Xaa-Pro dipeptide from the N-terminus of oligo- and poly-peptides [4]. Two types of DPP-4 are found in the body: soluble (sDPP-4) and membrane-bound (mDPP-4) forms that are only different in the presence or absence of cytoplasmic region at the N-terminal [5]. sDPP-4 is secreted from bone marrow, which can activate T-cell proliferation and may be involved in self/non-self-recognition [6,7]. In addition, mDPP-4 is located on the outside of the cell membrane in the liver, spleen, lung, brain, heart, and vascular smooth muscle cells [8]. The biological role of m DPP-4 is associated with insulin sensitivity regulation by degrading incretin like glucagon-like peptide 1 (GLP-1), which stimulates insulin biosynthesis, inhibits glucagon secretion, slows gastric emptying, reduces appetite, and stimulates regeneration of pancreatic β-cells [9]. Accordingly, DPP-4 inhibition becomes a novel approach to overcome insulin insensitivity associated with TII DM. Currently, synthetic DPP-4 inhibitors like sitagliptin and vildagliptin have clinically been used to treat TII DM. Interestingly, researchers have also reviewed current DPP-4 inhibitors as interacting with diuretics, warfarin, digoxin, and monoamine oxidase inhibitors, which alters DPP-4 metabolism or vice versa [10]. However, all synthetic DDP-4 compounds may occasionally contain adverse- or side-effects, and herbal medicines are believed to have lower adverse effects with similar efficacy [11]. Thus, scientists and physicians have paid more attention to the impact of searching for novel DPP-4 inhibitors from natural sources to provide new insights for TII DM patients.

Generally speaking, docking is a simulation of ligand–receptor interaction using computational algorithms [12]. Compared to other screening methods like enzymatic assay or in vitro cell assay, molecular docking can collect ligand candidates from a huge amount of compounds, which accelerates the progression of drug development [13]. In our previous study, 68 natural compounds were docked with DPP-4 protein module and the strength of interaction ranked by docking score. 16-hydroxycleroda-3,13-dien-15,16-olide (HCD) from *Polyathia longifolia*, rutin and quercetin from *Toona sinensis*, and berberine from *Coptis chinensis*, whose docking scores were 79.257 (rutin), 68.290 (HCD), 65.341 (quercetin), and 65.248 (berberine), respectively, obtained the 1st, 3rd, 5th, and 6th places of 68 screened natural compounds [14]. The data revealed that HCD, rutin, quercetin, and berberine might strongly interact with DPP-4 and which might be potential inhibitors. This study examined the inhibitory potency of selected DPP-4 inhibitor candidates through in vitro methods and further tested their hypoglycemic efficacy via short-term and long-term administration in vivo.

## 2. Results

### 2.1. In Vitro Inhibition of DPP-4 Activity by Natural Compounds

**Figure 1:**
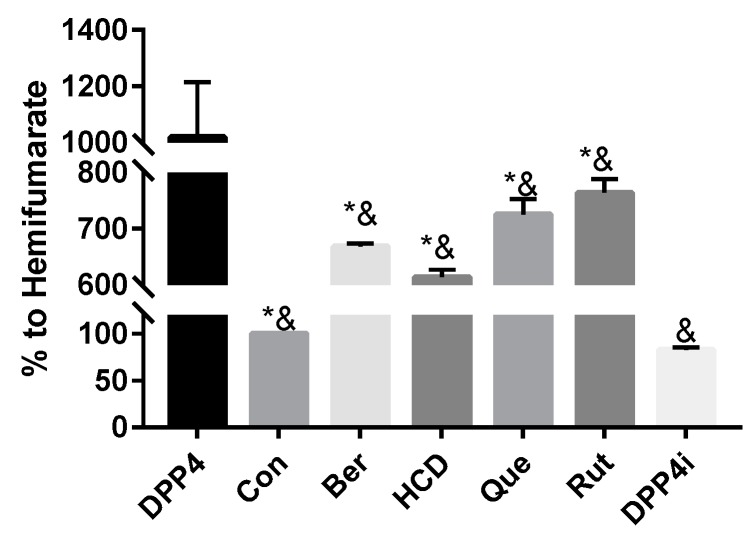
In vitro enzymatic assay of docked natural compounds against DPP-4. Natural compounds were mixed with DPP-4 and enzyme substrate and then the measured absorbance change at 405 nm. Result of DPP-4/hemifumarate (Con) was set at 100%. All data were represented as mean ± SD in triplicates from three independent experiments. * *p* < 0.05 was marked in the column significantly different with Con and ^&^ *p* < 0.05 with DPP4i. rutin (Rut), quercetin (Que), berberine (Ber), 16-hydroxycleroda-3,13-dien-15,16-olide (HCD).

To test which natural compounds directly inhibited the activity of the DPP-4 enzyme, 100 µM of rutin, quercetin, berberine, HCD, and two known DPP-4 inhibitors (Sitagliptin and hemifumarate) were used. When compared with original DPP-4 enzyme, all test samples showed an inhibition effect. The highest inhibition was found with HCD. Compared with hemifumarate, HCD showed higher inhibitory potency. Furthermore, compared with sitagliptin, the top three natural compounds showed lower (HCD and berberine) inhibitory potency than that of sitagliptin, which indicated natural compounds could reduce DPP-4 activity, but the inhibitory potency was not higher than clinical sitagliptin (Figure 1). Therefore, only the highest-potent candidate HCD was subsequently tested by an in vitro cellular assay.

**Figure 2:**
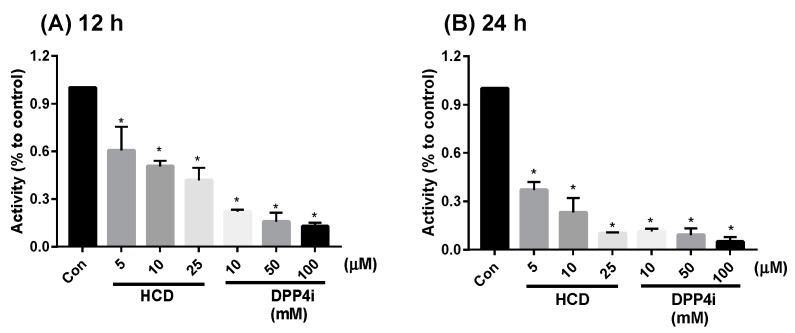
Alteration of Caco-2-bound DPP-4 activity by docked natural compounds. 16-hydroxycleroda-3,13-dien-15,16-olide (HCD) and sitagliptin (DPP4i) were treated with differentiated Caco-2 for (**A**) 12 h and (**B**) 24 h and DPP-4 activity determined. All data were converted into a ratio with the untreated control and shown as mean ± SD from three independent experiments. * *p* < 0.05 was marked in the column significantly different with Con.

On the other hand, mDPP-4 was a membrane-bound protein on the enterocyte, so DPP-4 activity inhibition of the two highest-potent compounds in enzymatic tests could also be determined by using enterocytic-mimic Caco-2 cells [15]. In 12 h and 24 h treatments, HCD showed inhibition in a dose-dependent fashion (Figure 2). However, the reducing fold of HCD was lower than sitagliptin. When the results were taken together, natural compounds selected by in silico could directly inhibit DPP-4 activity, but the inhibitory potency would not be higher than sitagliptin. Next, the inhibitory potency was evaluated at a cellular level.

### 2.2. Natural Compounds against DPP-4 Expression and Downstream Signaling Pathway

**Figure 3:**
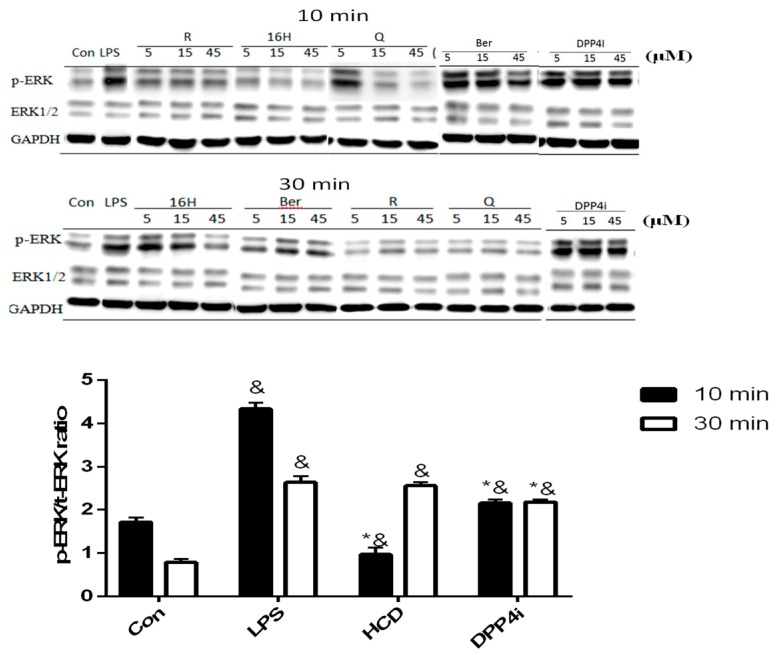
ERK phosphorylation change after selected natural compounds’ treatment. Myocyte were stimulated by LPS and then treated with, 16-hydroxycleroda-3,13-dien-15,16-olide (HCD & 16H) and sitagliptin (DPP4i) for 10- and 30-min. Ratio of phosphorylated and total ERK levels were detected by Western blotting and normalized with GAPDH. All data were mean ± SD from three independent experiments. * *p* < 0.05 was marked in the column significantly different to LPS and “&” with DPP4i.

Cellular DPP-4 has mDPP-4 and sDPP-4 as two forms, which act as different characters within cellular response regulation [16]. sDPP-4 could be a myokine that induces smooth muscle cell proliferation via up-regulating pro-inflammatory MAPK signaling pathway [17]. Thus, the inhibitory potency of DPP-4 in cellular level was determined via two different approaches: ERK-phosphorylation in smooth muscle cells and PKA expression in pancreatic cells. First, ERK-phosphorylation in LPS-induced smooth muscle cells could be used as a marker for intracellular DPP-4 activity. After 10 and 30 min of 10 ng/mL LPS stimulation, C2C12 cells were treated with three concentrations of natural compounds and ERK phosphorylation levels measured. These results were associated with enzymatic assay, all tested natural compounds could reduce ERK phosphorylation in C2C12 cells, which indicated that these compounds could block sDPP-4 activity (Figure 3). However, all concentrations of HCD except 45 μM showed no inhibitory effect in 30 min treatment, which was designated as the lower inhibition potency of these two compounds at higher inflammation levels (Figure 3).

**Figure 4:**
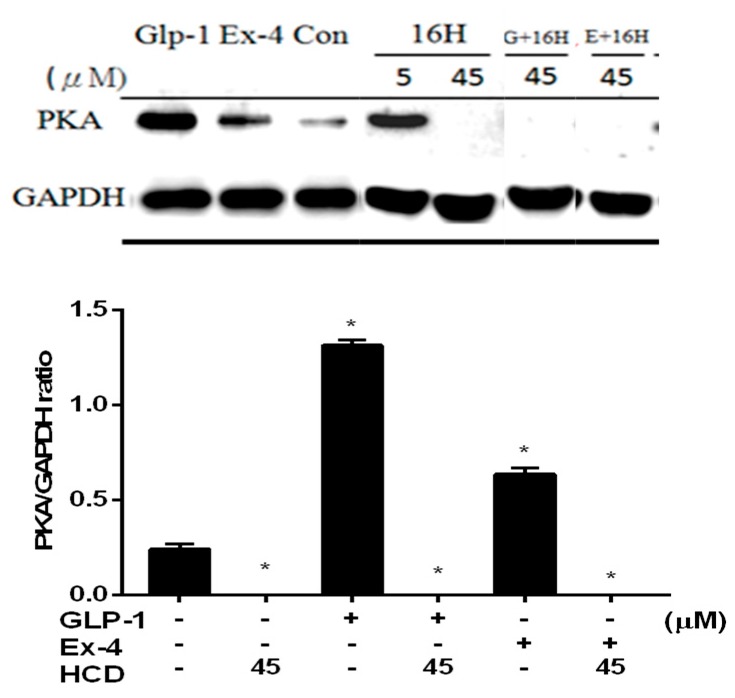
PKA level change after selected natural compounds’ treatment. Pancreatic cells were treated with and 16-hydroxycleroda-3,13-dien-15,16-olide (HCD & 16H) with/without GLP-1 (natural incretin) and exendin-4 (Ex-4, GLP-1 analogue) and PKA levels analyzed. PKA level was normalized with GAPDH and mean ± SD shown from three independent experiments. * *p* < 0.05 was marked in the column significantly different to the untreated control.

Moreover, mDPP-4 could be found in the pancreatic islet with the inhibition of up-regulated insulin secretion by PKA-dependent signaling [18,19]. The inhibitory potency of DPP-4 was measured by co-treatment with GLP-1 in pancreatic cells. PKA increased in GLP-1 and Ex-4 treated cells revealed a positive correlation between intracellular PKA and extracellular GLP-1. However, 45 μM of HCD treatment significantly blocked PKA expression. Even co-treating with GLP-1 and Ex-4 could not restore the PKA expression (Figure 4) Combining these data with the ERK-phosphorylation and DPP-4 inhibition results, HCD might not activate DPP-4 activity Therefore, this hindered that HCD strongly inhibited PKA expression through a signaling pathway other than GLP-1.

### 2.3. Single-Dose Hypoglycemic Effect of Natural Compounds

**Figure 5:**
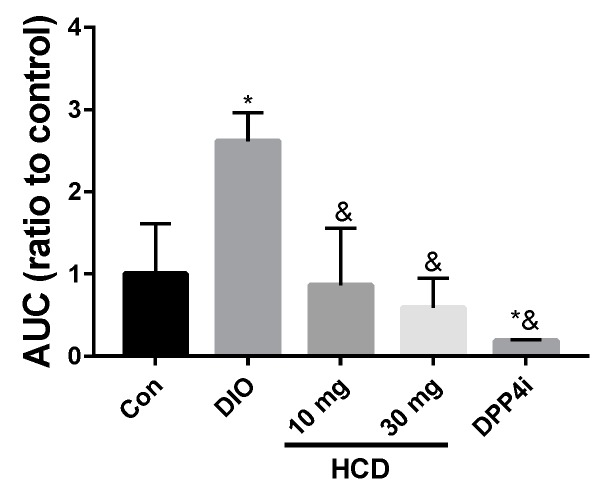
Insulin tolerance of DIO mice after selected natural compound gavaging. DIO mice were fed with HCD and Sitagliptin (DPP4i) and then insulin tolerance measured through OGTT. Results were shown as mean ± SEM from 6 individuals * *p* < 0.05 represented as significantly different compared with normal mice (Con), and ^&^ *p* < 0.05 represented as significantly different from DIO mice.

To understand the regulating effect of selected natural compounds on blood sugar in TII DM patients, diabetic DIO mice were administered HCD, quercetin, berberine, and sitagliptin (DPP4i) combined with 4 g/kg glucose to measure blood sugar changes. After converting blood sugar levels into the area under the curve (AUC), all treated groups showed a lower AUC than the DIO mice alone, which meant lowered blood sugar levels during the same testing period (Figure 5). Furthermore, the AUC of natural compound treated groups were not significantly different with normal mice. This result indicated that the tested natural compounds could safely lower blood glucose level but not cause a risk of hypoglycemia. When the above results were taken together, HCD could decrease blood sugar level in diabetic mice without increasing the risk of hypoglycemia. For further validation, the long-term control ability of HCD was tested.

### 2.4. Long-Term Blood Sugar Administration of Natural Compounds

**Figure 6:**
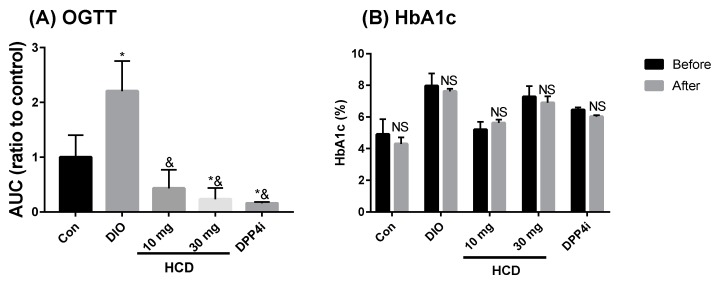
Insulin tolerance and glycated hemoglobulin (HbA1c) levels after long-term administration of natural compounds. DIO mice were fed with natural compounds for 5 weeks and insulin tolerance measured through (**A**) OGTT and (**B**) HbA1c levels. All results were mean ± SD from 6 individuals. * *p* < 0.05 meant significantly different as compared with Con (**A**) or before treatment (**B**), and ^&^ *p* < 0.05 represented significantly different from DIO mice.

Blood sugar control effects from natural compounds could be observed with two markers: AUC of the OGTT test and HbA1c %. After 28 days of natural compound feeding, AUC of DIO mice significantly ameliorated to be lower than the controls (non-induced) (Figure 6A). Compared with before and after sitagliptin (DPP4i), this result of HCD demonstrated that the hypoglycemic potential was similar to sitagliptin (DPP4i). However, HbA1c levels of HCD treatment were not different to these before treatment (Figure 6B), which hinders the reduction of advanced glycated end products in HCD and might be due to short duration of the compound and the cause of fluctuation of blood glucose. The next experiment was to evaluate the regulating efficacy of HCD in diabetic complications.

### 2.5. Dyslipidemia Reducing Effect in Long-Term Administration

**Figure 7:**
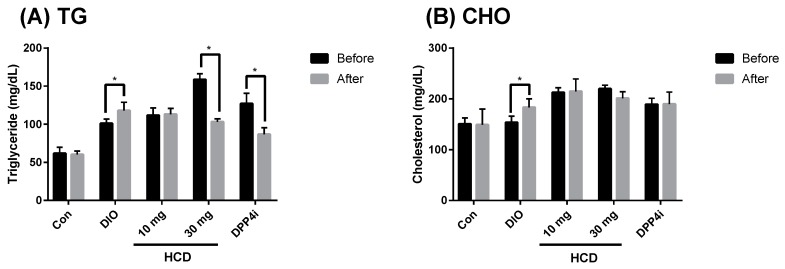
Alteration of serum lipid levels after selected natural compounds’ administration. HCD and sitagliptin (DPP4i) were fed to DIO mice for 5 weeks, respectively, and serum monitored (**A**) triglyceride and (**B**) cholesterol after treatment. All results were mean ± SD from 6 individuals. * *p* < 0.05 meant significantly different as compared with that before treatment.

In the pathology of diabetes, dyslipidemia has been well identified. In TII DM, dyslipidemia, especially hyperlipidemia and hypercholesterolemia, was not only induced by diabetes but also by a high fat diet [20]. Therefore, control of blood lipids and cholesterol could be a marker for diabetic control. After four weeks of treatment, total serum triglyceride (TG) and cholesterol were analyzed. When compared before and after the given natural compounds, serum TG in a high dose of HCD (30 mg/kg) treatment was significantly attenuated (Figure 7A). However, total cholesterol for each treatment was not different (Figure 7B).

### 2.6. Toxicity in Long-Term Administration

**Figure 8:**
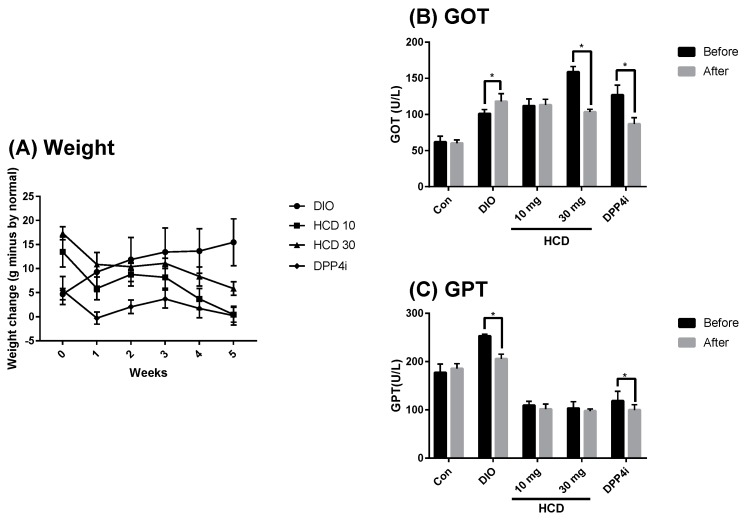
Estimating overall- and hepato-toxicity of selected natural compounds during long-term administration. Overall and hepatotoxicity in DIO mice fed with HCD or sitagliptin (DPP4i) for 5 weeks were monitored by (**A**) Body weight, (**B**) GOT, and (**C**) GPT. All results were mean ± SD from 6 individuals. * *p* < 0.05 meant significantly different as compared to that before treatment.

Although natural compounds were considered as having lower toxicity and less adverse effects than conventional agents, unsuspected toxicity in long-term administration still needs to be considered [21]. In this study, the body weight and GOT/GPT were used to monitor the overall and hepatotoxicity in HCD administration. When compared with untreated DIO mice, the body weight of HCD treatments was not significantly changed (Figure 8A). Furthermore, the GPT level after HCD treatment was significantly lower than before. These results implied that overall and hepatotoxicity were not observed (Figure 8B). Additionally, the GOT level of HCD treatment did not change when GPT decreased, indicating that liver inflammation might be ameliorated (Figure 8C). HCD exhibited hypoglycemic efficacy for short-term administration (not long-term), which implied the emergent use of HCD in rapid blood sugar control.

## 3. Discussion

To validate the DPP-4 inhibitory potency of HCD, berberine, rutin, and quercetin selected previously by in silico, the results of direct enzymatic alteration of selected natural compounds against DPP-4 showed inhibition effects in all compounds, with HCD being the most potent compound. In in vitro cellular assays, DPP-4-related activity and signaling change could also be observed in HCD treated in Caco-2 cells, LPS-activated ERK phosphorylation in myocytes, or PKA up-regulation in pancreatic cells. Using DIO-induced diabetic mice, HCD could decrease postprandial blood sugar regardless of single dose or long-term administration. However, HCD could not diminish HbA1c levels after long-term administration, which emphasizes HCD as not being able to decrease the highest level of postprandial blood sugar levels and eliminate blood sugar fast enough. Interestingly, HCD ameliorated DIO-induced hypertriglyceridemia and GOT activity after long-term administration. When taken together, HCD natural DPP-4 inhibitor selected from molecular docking is a compelling DPP-4 inhibitor and antidiabetic candidate.

After molecular docking, the binding pattern of selected natural compounds and DPP-4 were diagramed. Rutin formed three H-bonds at Glu^158^, Arg^252^, and Cys^257^; quercetin formed four H-bonds or π–π interactions at Arg^125^, His^126^, Glu^158^, and Phe^253^; HCD formed two H-bonds at Arg^125^ and Arg^186^; and berberine only formed one H-bond at Lys^55^ (Figure S1). However, comparing bond formation from simulation and in vitro enzymatic assay, bond formation seemed not to be related to the inhibitory activities, whereas HCD exhibited the highest inhibitory activity but did not form the most bonds in simulation (Figure 1). Besides, HCD and berberine interacted with Tyr^662^ and Tyr^666^ around the binding site and showed stronger inhibitory activities than those of rutin and quercetin. This impact could also be evaluated from the binding simulation of curcumin in a previous study [14]. This result speculated that interaction with Tyr^662^ and Tyr^666^ might be more important than others.

Screening DPP-4 inhibitors for treating diabetes is a recently developing field (since 2006), when a study reported that DPP-4 might regulate insulin sensitivity via degradation of GLP-1 [22]. Up to now, few natural DPP-4 inhibitors have been reported [23] From plant origin, DPP-4 inhibitory activities of kaempferol and derivatives, linustatins, chrysin, stigmasterol, emodin, lupeol, mangiferin, vitisins, and soybean hydrolysates were all validated via direct enzymatic assay and in vivo hypoglycemic analysis [24,25,26,27,28,29,30,31]. Moreover, the methanolic extracts of *Ficus benghalensis*, *Syzigium cumini*, *Ocimum sanctum*, and *Eucalyptus* sp. were also highlighted as sources of DPP-4 inhibitors [32,33]. From searching in animals, protein hydrolysates from milk and barbels with sequences of Leu–Pro–Val–Pro–Gln, Ile–Pro–Met, Trp–Ser–Gly, and Phe–Ser–Asp, elicited modifying activity for DPP-4 [34,35]. These reports emphasized the importance of searching for potent DPP-4 inhibitors from natural sources. Our study proposed HCD as a new DPP-4 inhibitor and further validated tits hypoglycemic efficacy in diabetic mice, which could be a new alternative hope for treating diabetes.

*Polyathia longifolia* is a febrifuge and indigestion drug in traditional Indian medication [36]. Recently, *P. longifolia* has been proven to have various biological activities such as anticancer, antimicrobial, immune-modulative, anti-hypertensive, and anti-ulcer [36]. Diterpene 16-hydroxycleroda-3,13-dien-15,16-olide (HCD) was isolated from *P. longifolia* bark with biological functions that pointed to two major phases: immunomodulation and anti-tumor [37]. Microglia could induce neuron cell death while it was stimulated by liposaccharide. HCD could down-regulate the expression of inflammatory-related signaling pathways including iNOS, COX-2, NF-κB, IκB, and TNF-α; and resulted in avoiding neuron cell death [38]. For the antitumor effect, HCD could activate apoptotic cell death in chronic myelogenous leukemia cells [39], renal carcinoma cells [40], autophagic cell death in oral squamous cell carcinoma [41], and glioma cells via ROS burst [42]. For breast cancer treatment, HCD pretreatment could potentiate tamoxifen tumoricidal efficacy via down-regulating anti-apoptotic signaling [43]. In renal cell carcinoma cells, HCD induced anoikis by decreasing focal adhesion kinase-related signaling, which blocks tumor cell invasion and turns into apoptosis [44]. A previous study reported the hypoglycemic activity of HCD via inhibiting α-amylase/α-glucosidase activity by the in-silico method [45,46]. This study afterwards elicited the hypoglycemic efficacy of HCD along with DPP-4 inhibition. Generally, the bioavailability of HCD via oral administration was low. Nanoparticle-encapsulation like mesoporous silica nanoparticles could significantly enhance the bioactivity of HCD in antidiabetic and antitumor activity [47,48].

In our previous study, rutin, quercetin, and berberine showed DPP-4 inhibitory potency while docking with DPP-4 [14]. In the literature, the hypoglycemic effect of berberine has been investigated for many years. Berberine could activate AMP-activated protein kinase (AMPK), nuclear factor erythroid-2-related factor-2 (Nrf2), and NF-κB signaling pathway and result in elevating insulin sensitivity [49]. Also, berberine could increase insulin secretion in vivo [49]. To the best of our knowledge, the hypoglycemic mechanism of rutin has been proposed in four directions: 1. elevation of glycogenesis and reducing gluconeogenesis; 2. promoting glucose uptake in myocytes by inducing translocation of glucose transporter 4; 3. reducing glucose absorption in the intestine; and 4. enhancing Langerhans islet cell activity and increasing insulin secretion [50]. For quercetin, the best-known hypoglycemic mechanism is α-amylase/α-glucosidase inhibition [45,46,51]. In this study, three natural-DPP-4 inhibitory candidates were investigated in an enzymatic and in vitro cellular assay, but their inhibitory potency was lower than for HCD. Additionally, other hypoglycemic mechanisms such as attenuating urine glucose reabsorption are worth further attention for testing.

## 4. Materials and Methods

### 4.1. Natural Compounds and Reagents

The molecular docking of natural compounds against DPP-4 was described in a previous study [14]. Selected natural compounds were purified by Yi-Chen Chia (Department of Food Science and Technology, Tajen University, Pingtung, Taiwan). Reagents and mediums for cell culture were obtained from Thermo-Fisher Inc. (Waltham, MA, USA). General chemicals were purchased from Sigma-Aldrich (Merck KGaA, Darmstadt, Hesse, Germany).

### 4.2. Western Blotting

Cells were collected and lysed in ice-cold RIPA buffer (150 mM NaCl, 1.0% NP-40, 0.5% sodium deoxycholate, 0.1% sodium dodecyl sulphate (SDS), 50 mM Tris-HCl at pH 8.0) with protein inhibitor cocktail (Roche Holding AG, Basel, Kanton Basel-Stadt, Switzerland) at 4 °C for 60 min. Cell debris was discarded by centrifugation at 12,000 *g* for 30 min at 4 °C, and protein concentration in suspension was quantified using a Bradford protein assay (Bio-Rad, Hercules, CA, USA). A total of 30 μg of proteins was separated using SDS-PAGE and, subsequently, transferred to a PVDF membrane (PERKin Elmer Life Sciences, Boston, MA, USA). Desired proteins were stained with appropriated 1st antibodies and HRP-conjugated 2nd antibodies. After staining, whole membranes were immersed in ECL reagent (Bio-Rad) and chemiluminescent intensity detected by a LAS-3000 imager (Fujifilm, Minato, Tokyo, Japan). Chemiluminescence of each protein was normalized with chemiluminescence of GAPDH, and the protein levels were presented as the intensity ratio to the untreated controls.

### 4.3. In Vitro DPP-4 Inhibitory Assay

#### 4.3.1. Cell Culture

Mouse myoblast cells C2C12, rat pancreas tumor cells AR42J, and human colorectal adenocarcinoma cells Caco-2 were obtained from the Bioresource Collection and Research Center (BCRC, Hsinchu, Taiwan) and American Type Culture Collection (Manassas, VA, USA), respectively. Cells were grown in high-glucose Dulbecco’s Modified Eagle’s Medium supplied with 10% (C2C12) and 20% (AR42J and Caco-2) fetal bovine serum plus 1% penicillin/streptomycin and the medium were changed every 2 days. Cells were maintained under 37 °C, 5% CO_2_ in water-jacket incubator (Thermo-Fisher) and detached by 0.25% trypsin/EDTA while reached 80% confluence. All experiments were carried out within 10 passages for controlling uniformity and consistency.

#### 4.3.2. Enzymatic Inhibition Assay

In vitro enzymatic assay of DPP-4 was carried out by Enzo^®^ DPP-4/CD26 Assay Kit for Biological Samples (Enzo Life Sciences, Inc., Farmingdale, NY, USA) and following the protocol in the manual. Briefly, 50 μL of assay buffer, 20 μL of DPP-4, and 20 μL of test samples (natural compounds, sitagliptin, and hemifumarate) were sequentially mixed in 96-well plate, respectively. After 30 min of incubation, 10 μL of enzyme substrate (H–Gly–Pro–pNA) was added and the optical intensity measured at 405 nm. The DPP-4 activity treated with hemifumarate was set as 100%, the tested samples were measured with hemifumarate and the data presented as the ratio to hemifumarate.

#### 4.3.3. Cellular DPP-4 Inhibition Assay

In vitro cellular DPP-4 inhibition assay could be divided into 3 assays: ERK phosphorylation inhibition in LPS-stimulated muscle cells, PKA activation in incretin-induced pancreatic cells, and expression inhibition in Caco-2 cells. The inhibition of DPP-4 expression in Caco-2 cells was performed by Western blotting. Caco-2 cells were inoculated into a 12-well plate followed by natural compounds’ treatment for 12, 24, and 36 h, respectively. After treatments, DPP-4 levels and PKA activating inhibition were determined by Western blotting. A number of 1 × 10^5^ cells/wells of AR42J cells was seeded into a 12-well plate and incubated overnight for confluence. Then, cells were incubated with 1 nM of GLP-1, exendin-4 (Ex-4), natural compounds with GLP-1, or natural compounds with Ex-4 for 48 h, respectively. Finally, PKA levels within AR42J were determined by Western blotting. LPS-induced ERK-phosphorylation followed the above protocol with slight modification. A number of 5 × 10^4^ cells/wells of C2C12 was used in this study. After incubating overnight, 10 ng/mL of LPS was added for 10 and 30 min, respectively, followed by culturing with natural compounds for 12 h. Phosphorylated-ERK was assayed by Western blotting.

### 4.4. In Vivo Animal Test

#### 4.4.1. Animals and Obese Induction

Six-week-old male ICR mice were obtained from the National Laboratory Animal Center (Taipei, Taiwan) and kept in controlled environmental conditions at room temperature (22 ± 2 °C) and with humidity (50 ± 10%). The 12 h light/dark cycle were maintained throughout the study. Mice had free access to food and water and were maintained on a standard laboratory diet. Animal experiments were approved by the National Dong-Hwa University Animal Ethics Committee (approval number 001 at 29 December 2014) and were used according to the “Guide for the Care and Use of Laboratory Animals” of National Dong-Hwa University. Diet-induced obese (DIO) mice were treated with a high-fat and high-fructose diet. ICR mice were divided into 2 groups: DIO mice were fed with high-fat (commercial diet supplied with 150 g/kg lard) and high fructose (60% *w*/*v*) diet for 14 weeks; control group was fed with a normal diet. At week 10, mice were treated with an oral glucose tolerance test (OGTT) and an insulin intolerance test for the determination of DM type. If the blood sugar level at 120 min after gavaging was higher than 200 mg/dL, mice were categorized as insulin intolerant. When the insulin-intolerant mice failed the insulin intolerance test, the mice could be diagnosed as TII DM mice.

#### 4.4.2. Oral Glucose Tolerance Test (OGTT) and Insulin Intolerance Test

To check diabetic symptoms in DIO mice, we used postprandial blood sugar level and insulin tolerance. Before operating OGTT, mice were fasted for 12 h, blood was sampled from the tail vein, and the blood sugar level was tested using an Accu-Chek blood sugar analyzer (Roche Holding AG) prior to oral gavage (p.o.) with 4 g/kg glucose solution. After p.o., blood sugar was continuously measured at 30, 60, 90, and 120 min after gavaging. All data in each group were collected and plotted with gavaging time to calculate the area under curve (AUC). Insulin intolerance testing used the same protocol with OGTT. After p.o. glucose, mice were immediately intraperitoneal (i.p.) injected 0.8 IU/kg Bwt insulin solution (Novo Nordisk A/S, Kalundborg, Denmark).

#### 4.4.3. Single-Dose Hypoglycemic Efficacy

To determine hypoglycemic efficacy in a single dose, OGTT in DIO mice was employed. OGTT was tested on DIO mice with the same protocol as in the previous section. After p.o. glucose, mice were immediately treated p.o. with natural compounds and blood sugar levels tested.

#### 4.4.4. Long Term Administration

Long term administration was carried out in DIO mice. Diabetic DIO mice were allotted to treated groups for 6 mice and treated p.o. with natural compounds once per 7 days for 28 days. Body weight was measured every 7 days. At the end of treatment, OGTT was carried out to check insulin tolerance.

#### 4.4.5. Blood Biochemical Detection

At the beginning and end of treatment, 500 µL of whole blood was collected by piercing the cheek and separating into 2 parts: 100 µL mixed with 3.2% sodium citrate to measure glycated hemoglobulin (HbA1c) immediately; 400 µL incubated at room temperature for coagulation and then centrifuging at 4000 rpm for 10 min to collect serum using total triglyceride (TG), total cholesterol (CHO), glutamate oxaloacetate transaminase (GOT), and glutamate pyruvate transaminase (GPT) tested by an automatic analyzer (ARTAX Menarini Diagnostics, Florence, Italy).

### 4.5. Statistical Analysis

All data were expressed as means with standard deviations (mean ± SD) and the data were analyzed using one-way ANOVA with a Dunnett’s test. The level of statistical significance was set at *p* < 0.05. All statistical procedures were performed with GraphPad Prism version 7.0 (GraphPad Software, Inc., La Jolla, CA, USA).

## 5. Conclusions

When taken together, HCD is first evident in showing DPP-4 inhibitory activity and blood sugar lowering efficacy in short-term and long-term administration. Combining with α-amylase/α-glucosidase, inhibition potency having been previously demonstrated, HCD poses a high potential for becoming an antidiabetic drug candidate. The antidiabetic potency of HCD has been under-estimated in research and it worthwhile seeking the impact of a clinical trial.

## Supplementary Materials

Supplementary materials can be found at http://www.mdpi.com/1422-0067/20/3/530/s1.

## Abbreviations

AUC	Area under curve
CHO	Total cholesterol
DIO	Diet-induced obese
DPP-4	Dipeptidyl peptidase 4
ECL	Enhanced chemiluminescence
ERK	Extracellular signal–regulated kinases
GAPDH	Glyceraldehyde 3-phosphate dehydrogenase
GLP-1	Glucagon like peptide 1
GOT	Glutamic oxaloacetic transaminase
GPT	Glutamic-pyruvic transaminase
HbA1c	Glycated hemoglobin
HCD	16-hydroxycleroda-3,13-dien-15,16-olide
HRP	Horseradish peroxidase
LPS	Lipopolysaccharide
OGTT	Oral glucose tolerance test
PKA	Protein kinase A
PVDF	Polyvinylidene difluoride
TG	Triglyceride
TII DM	Type 2 diabetes mellitus
