# Supplementary material for: Polyalthia Clerodane Diterpene Potentiates Hypoglycemia via Inhibition of Dipeptidyl Peptidase 4
Source: Int J Mol Sci. 2019 Jan 27;20(3):530. doi: 10.3390/ijms20030530 (PMC6387447; doi:10.3390/ijms20030530)
Supplement: Supplementary file 1 [file ijms-20-00530-s001.pdf]

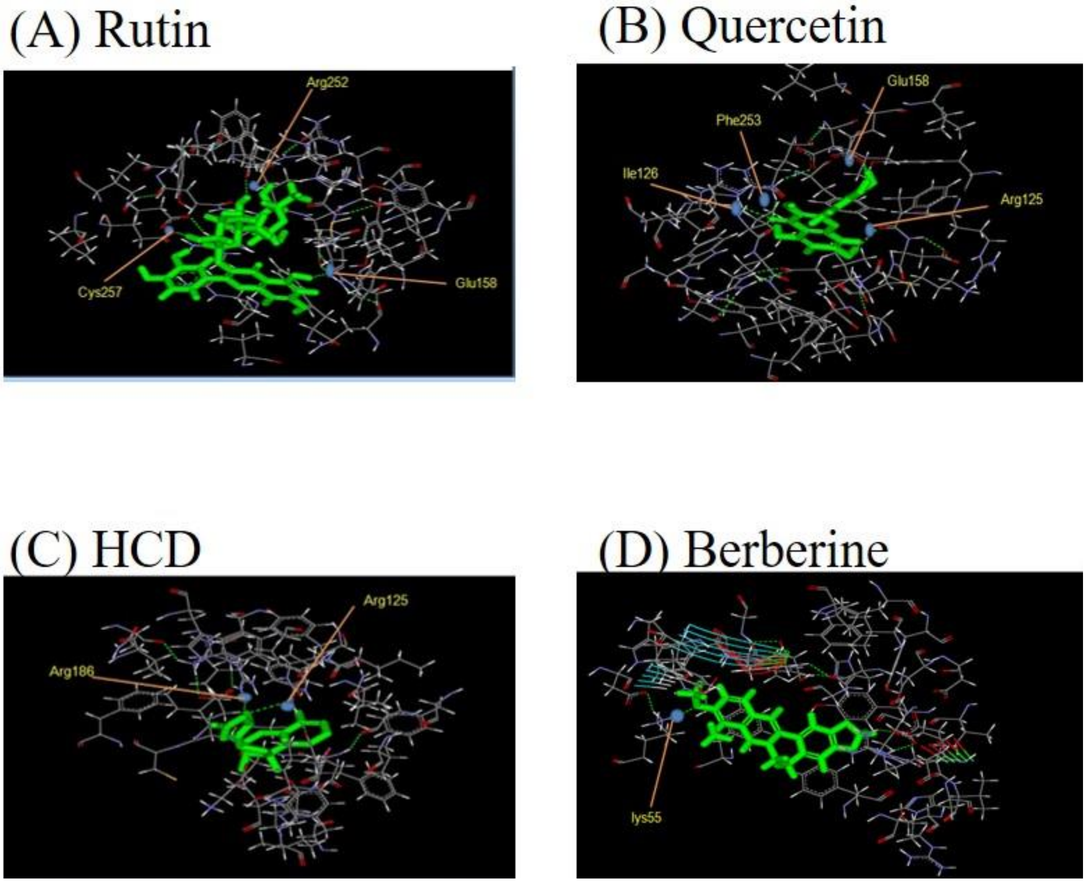

Figure S1. Binding diagram of selected natural compounds with DPP-4.
